# Recurrent Human Rhinovirus Infections in Infants with Refractory Wheezing

**DOI:** 10.3201/eid1506.081558

**Published:** 2009-06

**Authors:** Piyada Linsuwanon, Sunchai Payungporn, Rujipat Samransamruajkit, Apiradee Theamboonlers, Yong Poovorawan

**Affiliations:** Chulalongkorn University, Bangkok, Thailand

**Keywords:** Human rhinovirus, infants, refractory wheezing, respiratory infections, viruses, letter

**To the Editor:** Respiratory infections frequently cause illness among pediatric patients worldwide. Human rhinovirus (HRV) is a cause of acute respiratory tract infections (RTIs) (*1*); co-infections with other respiratory viruses such as respiratory syncytial virus (RSV) or influenza virus have also been reported. HRV strains have been subdivided into 2 genetic subgroups (HRV-A and HRV-B); a third genetic subgroup has been recently discovered (*2**–**7*). However, understanding of the epidemiology of novel HRV infection among Asian pediatric patients with respiratory illness (*4**,**5*) and association with recurrent wheezing and asthma has been limited (*8**–**10*).

We retrospectively analyzed 289 nasopharyngeal aspirates (NPAs) obtained from 286 pediatric patients admitted to Chulalongkorn Memorial Hospital in Bangkok, Thailand, during 2006–2007. The study was reviewed and approved by the Institutional Review Board, Chulalongkorn University, Bangkok, Thailand. Each specimen was tested for common respiratory viruses such as RSV, HRV, parainfluenza 1–3, influenza A and B viruses, adenovirus, human metapneumovirus, and human bocavirus. On the basis of phylogenetic analysis of the VP4 region, we identified 2 patients who had been admitted with 5 episodes of acute RTIs and subsequent recurrent wheezing associated with HRV-A and HRV-C.

The first patient was an infant girl whose first episode of breathing difficulty was at 5 months of age; a diagnosis of RSV bronchiolitis was made. She was hospitalized with respiratory failure and required mechanical ventilation for 3 days. At 6 months, she had pneumonia and wheezing. At 14 months, she had a low-grade fever, mild cough, breathing difficulty, and wheezing. While she was hospitalized for 7 days, a novel HRV-C (FJ435240) was identified by seminested PCR, and RSV was detected by reverse transcription–PCR. Seven months later, she had recurrent wheezing and respiratory distress. Virologic analysis indicated that she was co-infected with a divergent HRV-C strain (FJ435256) and influenza A virus. Nucleotide sequence identity score between the 2 isolated strains of HRV-C indicated a different cluster (identity score 70.1%).

The second patient was an infant boy with a diagnosis of acute bronchiolitis at 7 months of age. His underlying condition was congenital heart disease and an allergy to cow’s milk protein. Initial NPA showed HRV-A (FJ435274) and RSV by reverse transcription–PCR. Two months later, he had viral pneumonia and acute exacerbation of his reactive airway disease. He received systemic corticosteroids and a nebulized bronchodilator. His clinical course was complicated by 3 episodes of supraventricular tachycardia that were controlled with adenosine and cordarone. NPA was again positive for HRV-A (FJ435284). Three weeks later, he had upper respiratory tract symptoms, low-grade fever, and protracted cough; blood oxygen saturation was low and respiratory distress had rapidly increased. An NPA showed HRV-C (FJ435299). He received systemic corticosteroids and was discharged with corticosteroid inhalation. Comparison between 2 HRV-A strains isolated showed 82.5% nucleotide sequence identity. The sequence of the HRV-C strain also displayed 51.2% and 61.1% nucleotide identity to FJ435274 and FJ435284, respectively. Results of phylogenetic analysis are shown in the Figure.

**Figure:**
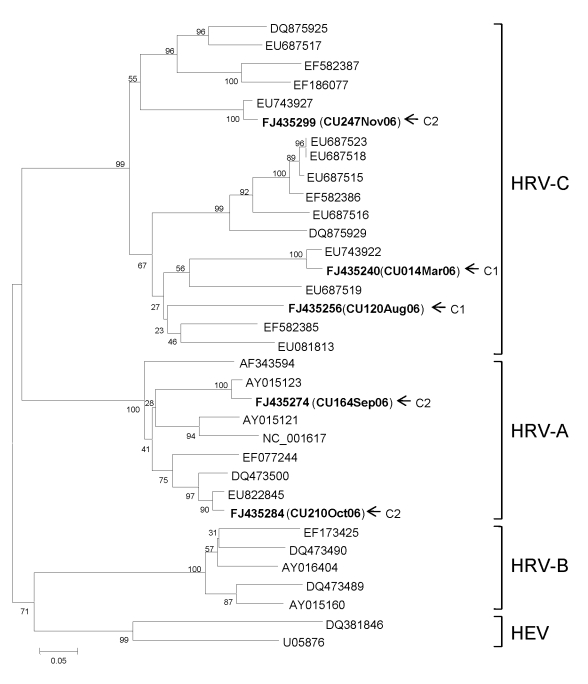
Phylogenetic analysis of nucleotide sequences of the virus capsid protein (VP4) region of 5 human rhinovirus (HRV) strains (shown in **boldface**) isolated from 289 nasopharyngeal aspirate specimens, including those of 2 infants with refractory wheezing (C1 and C2), on the basis of amplification of VP4/2 by seminested reverse transcription–PCR. The tree was constructed by using the neighbor-joining method and Kimura’s 2-parameter distance with bootstrap replicated from 1,000 trees by using MEGA 4.0 (www.megasoftware.net). Scale bar indicates number of nucleotide substitutions per site. Human enterovirus (HEV) was used as an outgroup for comparison.

A novel HRV-C infection in association with acute lower RTI was diagnosed in the first patient during her fourth and fifth hospitalizations. The 2 strains isolated are within the same genetic group and display 70% nucleotide similarity, which suggests that this infant was infected with 2 different virus strains. The second patient was infected with HRV-A during his first hospitalization. His condition subsequently progressed to refractory wheezing. Both patients were co-infected with RSV when a diagnosis of infection with lower RTIs was made. Two HRV-A strains detected in the second patient were within the same subgroup, but similarity in nucleotide sequences was only 82.5%. This result suggests that this patient was infected with 2 different virus strains of HRV-A and a strain of HRV-C.

Comparison of the HRV-A strains with the HRV-C strain showed that they belonged to different subgroups and had low similarity for nucleotide sequences. The second patient had 3 distinct rhinovirus infections over 3 months, and each was associated with illness requiring hospitalization. Both patients had underlying diseases, reactive airway diseases, and repeated episodes of RTI that may have rendered them vulnerable to reinfection, compromising their immune responses.

Complete coding sequences of HRV-A and HRV-C have been determined (*4**,**7*). However, little is known about their involvement in the pathogenesis of recurrent wheezing in young children. According to recent reports, HRV-C has been detected in hospitalized children with lower RTI in the People’s Republic of China (*5*). Possible association of novel infection with HRV and exacerbation of asthma in children has also been reported (*6*). We report HRV-A and HRV-C co-infections in conjunction with other respiratory viruses, such as RSV, as a potential cause of recurrent wheezing in infants with acute lower RTIs. Co-infections with HRV-A and HRV-C may contribute to increased virulence and subsequent pathogenesis of other respiratory viruses. Additional studies will be required to further explore the clinical role of novel HRVs.
